# Combining Thin-Section Coronal and Axial Diffusion Weighted Imaging: Good Practice in Middle Ear Cholesteatoma Neuroimaging

**DOI:** 10.3389/fneur.2021.606692

**Published:** 2021-09-07

**Authors:** Camilla Russo, Antonella Miriam Di Lullo, Elena Cantone, Michele Klain, Gaetano Motta, Andrea Elefante, Michele Cavaliere

**Affiliations:** ^1^Dipartimento di Scienze Biomediche Avanzate - Università degli Studi di Napoli “Federico II”, Naples, Italy; ^2^Dipartimento di Neuroscienze, Scienze Riproduttive e Odontostomatologiche - Università degli Studi di Napoli “Federico II”, Naples, Italy; ^3^CEINGE - Advanced Biotechnology, Naples, Italy; ^4^Dipartimento di Scienze Anestesiologiche, Chirurgiche e dell'Emergenza - Università degli Studi della Campania “Luigi Vanvitelli”, Naples, Italy

**Keywords:** middle ear, cholesteatoma, magnetic resonance imaging, diffusion weighted imaging DWI, fusion imaging

## Introduction

Along with clinical and otoscopic examination, magnetic resonance imaging (MRI) was proved to be the golden standard to assess the presence of cholesteatoma within temporal bone, both in middle ear cavity and mastoid. In particular, due to its specific composition (a cystic keratin-filled core surrounded by stratified squamous epithelium), cholesteatoma can be easily documented on diffusion-weighted imaging (DWI) as an area of striking hyperintensity due to restricted water diffusion (1, 2). Indeed, although computed tomography (CT) better defines localization and extent of the inflammatory tissue as well as the possible presence of bone erosions, only MR-DWI is able to define the nature of middle ear cavity opacification (cholesteatoma vs. granulation tissue) (3). Moreover, in recent times the extrapolation of quantitative values on apparent diffusion coefficient (ADC) maps generated from DWI has been proposed as a tool for distinguishing cholesteatoma from other types of middle ear inflammatory disorders (i.e., non-cholesteatomatous granulation tissue and abscesses), as well as to assess the risk of recurrence after surgical removal (4–7).

However, when referring to DWI for the assessment of skull base disorders such as cholesteatoma, it should be noted that a variety of different techniques could be applied (ranging from traditional spin-echo echo-planar images—EPI—to the more recently developed fast spin-echo–based non-EPI). Although these techniques are based on similar diffusion encoding, non-EPI ones differ in terms of image acquisition allowing for higher spatial resolution and lower susceptibility artifacts at air-bone interfaces (8–15).

Indeed, MRI protocol for hearing loss and to rule out the presence of cholesteatoma is generally based on the combination of sequences for brain imaging (generally including standard axial DWI, by far the most frequently used is spin-echo echo-planar due to its short imaging time and good contrast resolution) and specific sequences for the temporal bone (including thin-section coronal fast spin-echo–based non-EPI DWI, with some differences across sequences depending on MR unit vendor) (16); these latter are always acquired with small field of view (FOV), maximum section width of 3 mm and minimal or no inter-slice gap (1).

With this background, the aim of this retrospective study is to critically revise the role of combined thin-section coronal fast spin-echo–based non-EPI DWI of the temporal bone and axial spin-echo echo-planar DWI for the detection of middle ear cholesteatoma (both in the setting of acquired and residual/recurrent disease), in order to improve clinical management and optimize surgical procedures (17, 18).

## Materials and Methods

We retrospectively analyzed all patients with clinical suspicion of unilateral middle ear cholesteatoma who underwent MRI at our University Department between January 2010 and January 2020; both acquired and residual/recurrent disease were considered for our purposes, whereas no case of congenital cholesteatoma was included in the study. Cholesteatoma diagnosis was then confirmed at surgery. MRI was performed on the same 1.5 T unit (Philips Intera, Philips Medical Systems, Netherlands) with an 8-channel head coil. MRI protocol for hearing loss was based on sequences for whole brain imaging (generally including standard axial DWI) and specific sequences for the temporal bone (generally including thin-section coronal DWI). Standard MRI examination did not routinely include intravenous injection of gadolinium-based contrast media; patients with hearing implants and motion artifacts at MRI examination were excluded from the analysis.

DWI sequences parameters were set as follow:

- Brain axial spin-echo EPI DWI: 24 slides; TR 2,800 ms; TE 75 ms; thickness 5.00 mm; inter-slices gap 5 mm; FA 90; view size 2,338 × 1,228; matrix 128 × 128; *b* = 0, *b* = 500, and *b* = 1,000 s/mm^2^; 4 averages;
- Thin-section coronal multi-shot (MSH) non-EPI DWI of the temporal bone: 20 slides; TR 3,000 ms; TE 82.44 ms; thickness 3.00 mm; inter-slices gap 0; FA 90; view size 1,168 × 1,230; matrix 152 × 152; *b* = 0 and *b* = 800 s/mm^2^; 5 averages; cardiac gating to limit patient-related artifacts due to heart pulse and blood flow.

Finally, 173 MRI examinations were reviewed (97 female; 76 males; mean age 44.3 y; range 18–83 y), of them 89 for newly diagnosed acquired middle ear cholesteatoma (51.5%) and 84 for residual/recurrent disease (48.5%). Few patients (*n* = 9) had only axial DWI, as cholesteatoma discovery was an incidental finding on brain MRI performed for different diagnostic purpose; of the remaining subjects, 62 patients had only thin-section coronal DWI, whereas 102 had both axial and thin-section coronal DWI. Therefore, 111 axial DWI (54 newly diagnosed acquired and 57 residual/recurrent cholesteatomas) and 164 thin-section coronal DWI (80 newly diagnosed acquired and 84 residual/recurrent cholesteatomas) were globally revised; localizations of restricted water diffusion areas suggestive for cholesteatoma were noted separately on both axial and thin-section coronal DWI by two experienced neuroradiologists in consensus.

For thin-section coronal non-EPI DWI, axial EPI DWI, and combined thin-section coronal and axial DWI, diagnostic accuracy (DA), sensitivity, specificity, negative predictive value (NPV) and positive predictive value (PPV) were computed. All statistical analysis was performed using XLSTAT software (v.2019.1).

## Results

Standard axial EPI DWI alone showed an overall DA of 0.66 in identifying cholesteatomatous tissue within middle ear cavity, with 0.79 sensitivity, 0.32 specificity, 0.75 PPV and 0.37 NPV.

Thin-section coronal non-EPI DWI of the temporal bone had an overall DA of 0.73, with 0.83 sensitivity, 0.60 specificity, 0.71 PPV, and 0.75 NPV. Finally, combining axial brain EPI DWI and thin-section coronal non-EPI DWI of the temporal bone, DA increased up to 0.94, with 0.98 sensitivity, 0.94 specificity, 0.98 PPV, and 0.94 NPV.

Moreover, when separately analyzing results from the subgroups, it was found that the impact of the combination of the two acquisition plans was higher for residual/recurrent disease rather than for primary acquired middle ear cholesteatoma. Indeed, for primary acquired cholesteatoma standard axial EPI DWI showed a DA of 0.71 whereas thin-section coronal non-EPI DWI showed a DA of 0.88; combining the two acquisition techniques, DA increased to 0.98. Conversely, for recurrent/residual disease standard axial EPI DWI showed a DA of 0.52 and thin-section coronal non-EPI DWI showed a DA of 0.68, whereas with the combination of the two acquisition techniques DA increased to 0.90. The magnitude of the improvement was therefore higher in the second group compared to the first one.

Inter-observer reliability regarding cholesteatomas' identification, assessed with Cohen's kappa, was found at 0.94.

**Figure 1:**
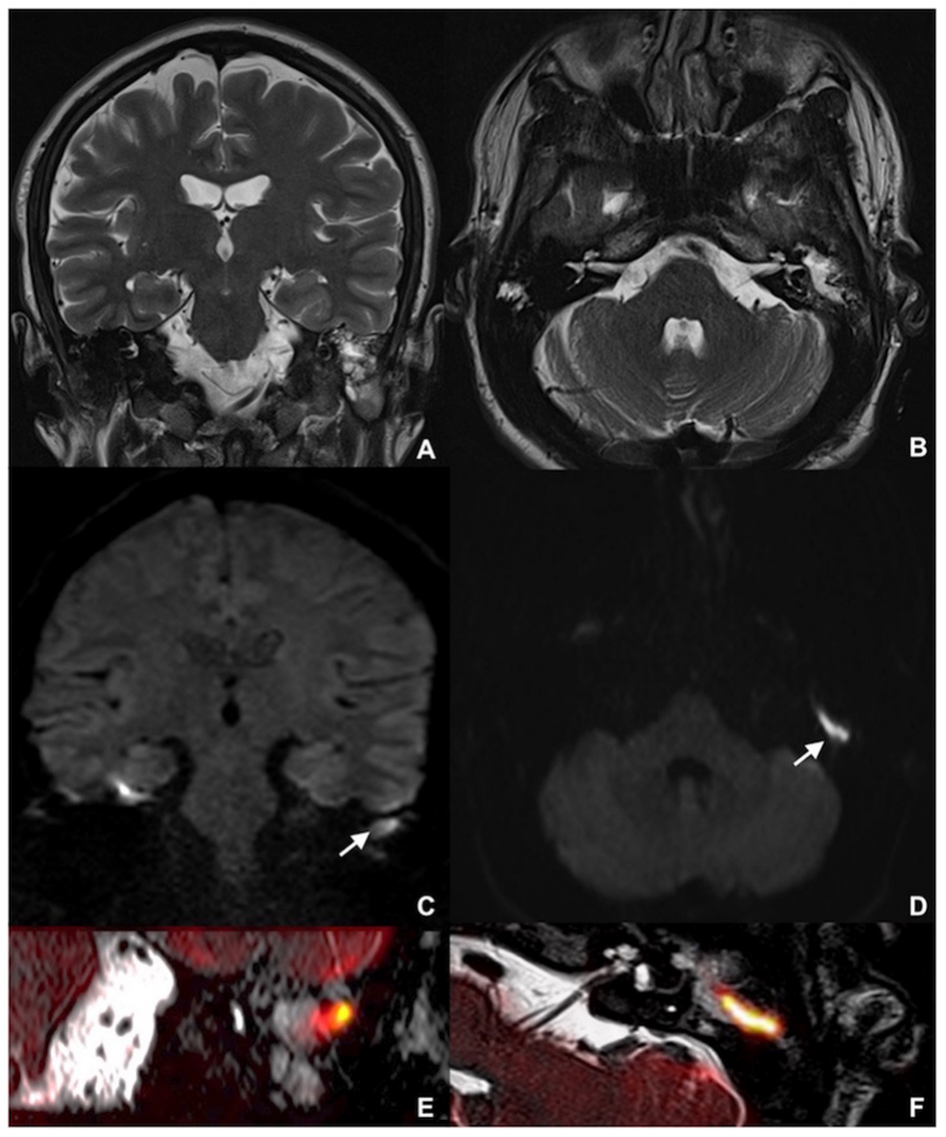
MRI showing left middle ear recurrent cholesteatoma in a 26-year-old man: coronal **(A)** and axial **(B)** TSE T2w; coronal MSH non-EPI DWI **(C)** and axial SE-EPI DWI **(D)**; detailed coronal **(E)** and axial **(F)** fusion imaging of both ultra-thin heavily-weighted 3D T2w sequences and DWI images. MRI, magnetic resonance imaging; TSE, turbo spin echo; MSH, multi-shot; EPI, echo planar imaging; DWI, diffusion weighted imaging; SE, spin-echo.

Overall results in terms of diagnostic testing accuracy are listed in Table 1. An example of axial and coronal DWI, along with fusion imaging with ultra-thin heavily-weighted 3D T2w sequences to allow for a better localization of cholesteatomatous tissue, is shown in Figure 1.

**Table 1:** Table resuming diagnostic testing accuracy (DA) for all cholesteatomas (*first column*), primary acquired cholesteatomas (*second column*), and residual/recurrent cholesteatomas (*third column*).

	**All**	**Primary**	**Residual/**
	**cholesteatomas**	**acquired c**.	**recurrent c**.
Axial EPI DWI	0.66	0.71	0.52
Coronal non-EPI DWI	0.73	0.88	0.68
Combined DWIs	0.94	0.98	0.90

## Discussion

DWI has become a pivotal MRI technique for the detection of middle ear cholesteatoma, both in case of newly diagnosed acquired lesions and post-surgical evidence of residual/recurrent disease at longitudinal follow-up (10, 19). In particular, thin-section coronal fast spin-echo-based non-EPI techniques have largely replaced traditional spin-echo EPI techniques for temporal bone imaging, due to their better spatial resolution with different acquisition matrix, lower signal-to-noise ratio, and reduced susceptibility artifacts at air-bone interfaces, thus allowing for cholesteatoma detection even when smaller than 5 mm (9, 20–22). These sequences include a variety of different techniques depending on MR vendor, ranging from half-fourier acquisition single-shot turbo spin-echo (SSH-TSE) to PROPELLER DWI, from BLADE to MSH non-EPI DWI; among them, the latter has been adopted in our MRI protocol for temporal bone as previously described (1, 8).

However, despite the good resolution and the lower incidence of interface artifacts in thin-section coronal fast spin-echo–based non-EPI techniques, few cases of false negatives and false positives have still been observed. While false negatives are mainly due to very small lesion size, false positives can be due to several different condition that could enhance the presence of MRI artifacts (i.e., presence of prosthetic materials or metallic dental implants, recent surgery, admixed otomastoiditis with areas of abscessualisation) (1, 23, 24). To overcome possible pitfalls and implement the interpretation of indeterminate single-plan DWI, additional axial DWI can be a worthwhile information support. In our experience, combining thin-section coronal non-EPI DWI and axial EPI-DWI improved the identification of small middle ear lesion, particularly in the setting of post-operative residual/recurrent disease where potential confounding factors can make proper and timely diagnosis more challenging.

Main limitations of the study are represented by the retrospective design and the moderate sample size; moreover, although similar diagnostic performances of different fast spin-echo–based non-EPI techniques were reported in literature, it should be considered that these observations apply to a specific non-EPI sequence and a single MR unit (despite probably representing the most common setting in daily clinical routine).

In conclusion, standard axial EPI-DWI combined to thin-section coronal fast spin-echo–based non-EPI DWI of the temporal bone has the potential to improve the diagnosis of middle ear cholesteatoma. This is all the more true when considering lesions smaller than 5 mm and/or residual/recurrent disease, when single plan DWI can be misleading and inconclusive. With this knowledge and due to its short acquisition time, we strongly recommend always including standard axial brain EPI-DWI along with thin-section coronal DWI in case of suspected cholesteatoma, in order to improve observers' confidence and enhance lesions' detection.

## Author Contributions

CR and AE conceived the study. AMDL and MC performed the analysis. GM and EC verified the analytical methods. MK supervised the findings of this work. All authors discussed the results and contributed to the final manuscript.

## Conflict of Interest

The authors declare that the research was conducted in the absence of any commercial or financial relationships that could be construed as a potential conflict of interest.

## Publisher's Note

All claims expressed in this article are solely those of the authors and do not necessarily represent those of their affiliated organizations, or those of the publisher, the editors and the reviewers. Any product that may be evaluated in this article, or claim that may be made by its manufacturer, is not guaranteed or endorsed by the publisher.

## Abbreviations

MRI: Magnetic Resonance Imaging
DWI: Diffusion Weighted Imaging
CT: Computed Tomography
ADC: Apparent Diffusion Coefficient
EPI: Echo-Planar Imaging
FOV: Field Of View
DA: Diagnostic Accuracy
NPV: Negative Predictive Value
PPV: Positive Predictive Value
SSH: Single-Shot
TSE: Turbo Spin-Echo
MSH: Multi-Shot.
